# Financial Vulnerability, Financial Literacy, and the Use of Digital Payment Technologies

**DOI:** 10.1007/s10603-022-09512-9

**Published:** 2022-03-07

**Authors:** M. M. Naeser Seldal, Ellen K. Nyhus

**Affiliations:** Department of Management, University of Agder, Postboks 422, 4604 Kristiansand, Norway

**Keywords:** Digital payment technologies, Mobile payment, Financial literacy, Financial vulnerability, Pain of paying

## Abstract

The purpose of this study is to test the notion that the use of digital payment methods, such as paying with a mobile phone, increases the risk of financial vulnerability. Research from the USA indicates such a relationship, and we study whether this finding can be generalized to other countries. Motivated by recent changes in EU legislation related to financial transactions, we also examine willingness to use social media companies for money transfers along with sharing bank account information with third-party financial services. Exploiting data collected from a representative sample of the Norwegian adult population (*n* = 2202), we identify differences in financial behaviour and characteristics between users and nonusers of different digital payment methods. In contrast to US studies, we find that mobile payment users were less financially vulnerable than nonusers and those women were more likely users of digital payment technologies than men. Younger generations and those with low financial literacy were more financially vulnerable than others, although we did not find this to be related to the use of mobile payment or other digital payment methods. The results show that there is a need for more research from different countries outside of the USA to obtain an understanding of the consequences of increased digitalization of financial services. In addition, as COVID-19 has shifted a vast amount of spending online and these newer payment technologies have become more available, we need to gain a better understanding of how they influence financial behaviour.

Recent studies have worryingly concluded that mobile payment users in the USA are at a higher risk of financial mismanagement than nonusers. In a world that moves quickly in the direction of forsaking the use of cash for digital financial tools (Mumtaza et al., 2020; Wewege & Thomsett, 2019), fueled even more by the COVID-19 pandemic (Shearman, 2020; eMarketer, 2021), it is important to investigate whether these US results are generalizable for other countries and generations. Gaining this knowledge could be imperative in taking measures to help stifle this financial mismanagement.

As new technological innovations are profoundly changing how financial services are conducted worldwide and are increasingly affecting financial behaviour (Gomber et al., 2017; Liu et al., 2020; OECD, 2017), new technologies could also potentially help consumers take more control over their personal finances (Wewege & Thomsett, 2019). However, the abovementioned studies from the USA suggest the opposite: the use of certain digital payment methods may increase financial vulnerability. Relatedly, in a recent study by Heo et al. (2021), respondents with a higher level of financial distress had a tendency to engage more in financial technology. The fact that these payment methods are both less transparent and more effortless to use than, e.g., cash and credit cards, could make some consumers more vulnerable to purchasing temptations. We therefore need more research on how consumers use these new technologies and their effects on financial behaviour. In particular, we need to study any negative side effects for consumers or specific consumer groups to inform service designers and regulators.

In this study, we analyze survey data from a representative sample of the Norwegian population to investigate whether we find the same relationships in a Northern European country with a population that has a higher level of financial literacy, is more digitally advanced than the US population,^1^ and is one of the countries furthest along in the world in becoming a cashless society (Mumtaza et al., 2020; Wewege & Thomsett, 2019). In this article, we include three different types of digital payment technologies, not just mobile payments as in US studies, and look at all adult generational groups. We also investigate which consumers are the most likely first adopters of the next generation of financial transaction technologies by identifying consumers who have positive attitudes toward sharing bank information with a third party, such as a social media company.

## Literature Review

The motivation for this and five recent US studies (Liao & Chen, 2020, 2021; Lusardi et al., 2017; Scheresberg et al., 2020; Yakoboski et al., 2018) was to acquire more knowledge about the consequences of the increase in use of new digital financial transaction services offered to consumers. The number of different types of digital payment solutions available to consumers, including solutions such as online payments (using payment systems for online transactions, e.g., PayPal, Klarna, Amazon Pay), mobile payments (using mobile payment apps, e.g., Payr, WeChat, Alipay, Venmo, Vipps), and contactless payments (by holding a payment card or smartphone over the terminal, using, e.g., Apple Pay, G Pay, Samsung Pay), has rapidly increased in recent years. Financial transactions are now increasingly performed by using these new digital technologies. Digital payment methods have many benefits due to their immediate availability and point of sale efficiency (Ozturk et al., 2017; Polasik et al., 2012). Transactions conducted through these new technologies are more convenient, easier, and quicker than the use of traditional technologies such as credit cards, checks, or cash. During the COVID-19 period, the use of these technologies was safer since payment did not involve touching cash or keyboards to enter codes.

Studies have shown that consumers spend more when using less transparent payment methods. Several studies have compared the use of credit cards or debit cards to cash (Feinberg, 1986; Hirschman, 1979; Runnemark et al., 2015), where card users have been found to spend more than people paying with cash. To explain the credit card premium, Zellermayer (1996) coined the term “pain of paying”, as the feeling consumers encounter when paying. The more transparent a mode of payment is, the greater the “pain of paying,” i.e., aversion to pay, with cash being the most transparent mode (Prelec & Loewenstein, 1998; Raghubir & Srivastava, 2008; Soman, 2003) and digital payment methods the least transparent (Shah et al., 2015). This idea that a decision to purchase entails a pain of paying is also consistent with neuroscientific evidence (Knutson et al., 2007). In a study using event-related fMRI to investigate how people make purchasing decisions, Knutson et al. (2007) found that consumers have a tendency to overspend when using a credit card rather than cash. Similar results were found in a study on the use of cash vs. credit cards when buying goods with uncertain market value—in this case, tickets to sporting events (Prelec & Simester, 2001). The delayed payment with the credit card along with its abstract nature could “numb” consumers against the pain of paying (Prelec & Loewenstein, 1998). Thus, this reduction in the use of cash in favour of less transparent payment modes may decrease control over spending (Agarwal et al., 2019; Meyll & Walter, 2019; Yakoboski et al., 2018). As digital payment methods are seen as some of the least transparent (Shah et al., 2015), adopting them might therefore lead to an additional decrease in spending control. Something strengthened by recent studies, where Liu et al. (2020) found that the use of mobile payments increased the willingness to pay (WTP) compared to using cash. Another study by Boden et al. (2020) found that the use of mobile payments can increase WTP compared to the use of credit cards; however, they linked this to convenience and did not find that the pain of paying was any different between the two payment technologies. Although it is not yet clear that there is a significant decrease in pain of paying when moving from paying with credit cards to paying with a mobile phone, this effect of the use of nontransparent payment methods should be further studied.

Currently, there is no universally accepted definition of household financial vulnerability (O'Connor et al., 2019). Even “financial vulnerability” as a term is used interchangeably with terms such as financial fragility (Ampudia et al., 2016), financial distress (Anderloni et al., 2012), financial debt burden (Poh & Sabri, 2017), and financial over indebtedness (Daud et al., 2019). Many different variables are applied to indicate financial vulnerability, but one aspect that seems present in all studies is debt, (Anderloni et al., 2012; Bankowska et al., 2017; Daud et al., 2019; Leika & Marchettini, 2017; Noerhidajati et al., 2021), and in many cases, unsecured debt (e.g., credit card debt and other consumer debt) (Anderloni et al., 2012; Fuenzalida & Tagle, 2009; Lusardi et al., 2020). This kind of debt makes a household particularly vulnerable to adverse shocks such as job loss, reduction in working hours, illness, and the death of a bread winner. Households are also presumed fragile and vulnerable if they are unable to pay their monthly expenses (Bridges & Disney, 2004). In contrast, having a savings buffer will ensure that a household is less financially vulnerable to these kinds of shocks (Gjertson, 2016; O'Connor et al., 2019). Researchers have also found a link between financial vulnerability and financial literacy, where higher levels of financial literacy along with financial education reduce financial vulnerability (Anderloni et al., 2012; Yusof et al., 2015). Relatedly, Heo et al. (2021) found that people with a greater level of financial stress were more likely users of financial technology. For the purpose of our study, we define financial vulnerability as the absence of a buffer for unexpected expenses, difficulties with making ends meet, and borrowing for consumer purchases.

Financial literacy has also been found to have an effect on financial behaviour (Lusardi & Mitchell, 2014), which makes it necessary to obtain an understanding of how people grasp basic financial concepts (Klapper et al., 2015). In 2004, Lusardi and Mitchell designed three questions to measure financial literacy, which have become a benchmark and are often referred to as the “Big Three” (Lusardi & Mitchell, 2011a, 2011b). The questions were incorporated into the 2009 U.S. National Financial Capability Study (NFCS), and along with two more questions from the study, they have become known as the “Big Five” (Hastings et al., 2013) and is the most prevalent measure of financial literacy. Strong relationships have been found between the level of financial literacy and paying bills on time, paying credit card bills in full each month (Hilgert et al., 2003), planning for retirement, and accumulating savings and wealth (Ameriks et al., 2003, Hung et al. 2009, Stango & Zinman, 2009, Lusardi & Mitchell, 2011a, 2011b, Van Rooij et al., 2011, Hauff et al., 2020). Studies have, e.g., linked low financial literacy to excessive debt accumulation (Lusardi & Tufano, 2015; Stango & Zinman, 2009), and having high-cost debt, debt problems and suboptimal mortgages is found to be more common among people with a low level of financial literacy (Moore, 2003, Disney & Gathergood, 2013, Lusardi and Scheresberg 2013, Lusardi & Tufano, 2015). A few recent studies also suggest that the combination of the use of digital payment methods and low financial literacy may have negative effects on financial behaviour. In a study on mobile payment users and credit card behaviour involving more than 25,000 US households, Meyll and Walter (2019) identified costly credit card behaviour to be more likely among mobile payment users. In addition, they found that users of this payment method were likely to be young, male, and have higher levels of education and income, while they were less financially literate than nonusers. Similarly, Lusardi et al. (2017), Yakoboski et al. (2018), and Scheresberg et al. (2020) found that the use of mobile payments was related to lower financial literacy. They linked the use of mobile payments to a higher likelihood of overdrafts and the extensive use of credit cards among Millennials (aged 22–37), along with finding that higher financial literacy reduced the negative effects of using mobile payments. In another US study, Garrett et al. (2014) found that the use of mobile payments was negatively related to financial knowledge and that mobile payment users displayed poor credit card behaviour and reported higher difficulty paying bills.

Millennials (born 1981–1996) have been the generation taking the lead in the adoption of new digital technology, and they are also the most studied generation regarding technological payments (e.g.Lusardi et al., 2017; Scheresberg et al., 2020; Yakoboski et al., 2018). However, we can also acknowledge that the use of digital financial technology among older generations has been growing significantly in recent years. For example, in the USA, 92% of Millennials (born 1981–1996), 85% of those belonging to Generation X (born 1965–1980), 67% of Baby Boomers (born 1946–1964), and 30% of those in the Silent Generation (born 1928–1945) owned a mobile phone in 2018 (Jiang, 2018). In Norway, the corresponding numbers for 2020 were 99% of Millennials, 97% of Generation X and 79% of Baby Boomers and Silent Generation (only measured up to 79 years old) (MEDIENORGE, 2021). Currently, we know little about the association between mobile payment methods and financial problems in other generational groups. We therefore include these older generational groups in this study.

In 2019, the new Payment Service Directive (PSD2) was implemented in the EU to increase competition and innovation within payment services. As a result, a new generation of digital financial services has been and will be made available to consumers. Many of these new technologies are less transparent than those already in use, something that might lead to some consumers being more financially vulnerable. For this reason, we want to investigate which groups are most likely to take advantage of these new technologies and whether some of these groups are more likely to be financially vulnerable.

When considering who the adopters of new digital payment technologies are, previous studies have found that they are likely to be young adults and those with high technological competence (Choudrie et al., 2018; Elhajjar & Ouaida, 2019; Meyll & Walter, 2019; Scheresberg et al., 2020; Yakoboski et al., 2018). A higher likelihood of adoption was found among men (Garrett et al., 2014; Laukkanen & Pasanen, 2008; Yeo & Fisher, 2017), people with higher education (Jünger & Mietzner, 2020), people with higher financial literacy (Jünger & Mietzner, 2020), and people from lower income households (Choudrie et al., 2018). At the same time, young adults have been found to be less financially literate than older generations, which means that the use of financial technologies does not necessarily imply financial understanding (Lusardi & Mitchell, 2011a, 2011b; Scheresberg et al., 2020; Yakoboski et al., 2018). Studies have also shown that loan defaults are growing among young people (Andersen, 2017, Dinero, 2019), and changes in the use of payment technologies may play a role in this development, especially since it implies a reduced use of cash (Humbani & Wiese, 2018; Kumari & Khanna, 2017).

To learn more about the advantages and disadvantages associated with this shift toward less transparent ways of paying, further studies are needed. In particular, it is necessary to know whether the users of new digital technologies have sufficient financial knowledge to understand the consequences of letting, for example, a social media company handle their financial transactions and whether the use of digital technologies may contribute to making consumers more financially vulnerable. This study contributes to the literature by including the use of more types of digital technologies than previous studies (mobile payments, contactless payments and online payments) and by exploring attitudes toward upcoming changes in the financial environment. This study also represents a replication of US studies on the effects of mobile payments in a country with a higher average level of financial literacy (Klapper et al., 2015; Lusardi & Mitchell, 2011a, 2011b) and a slightly higher degree of digitalization (Chakravorti et al., 2017; WEF, 2016).

We hypothesize that the use of digital payment technologies is linked to a higher probability of financial vulnerability, e.g., overspending on credit cards or using high-cost consumer debt, defaulting on bills, or not having enough savings for a rainy day. In line with the previously mentioned research from the USA, we also hypothesize that users of digital payment technologies have lower financial literacy than nonusers. We expect to find these relationships in all generations and for all digital payment technologies included in the study, particularly in the group “Millennials/Generation Z (born 1981–2012).” Furthermore, with regard to the participants’ financial literacy level and financial vulnerability, we expect to find similar relationships among those who are willing to let banks share their information and among those who are willing to use social media companies for money transfers. Additionally, we control for other demographic variables that previous research has identified as possible predictors of vulnerability (gender, age, education level, income level, and financial literacy).

The rest of this paper is structured as follows: The next section describes the methods used to collect the data and the measurement of the main variables. The results section presents the descriptive statistics of the sample and results. The theoretical and practical implications of the findings are discussed in the discussion section, while the final section concludes.

## Data

### Data Collection

For the purpose of this study, we exploited data collected from a representative sample of 2209 adult Norwegians who participated in an online survey administered by Kantar TNS in June 2018. There was an overrepresentation of men (53.8%) and the older population in the sample, and the data have been weighted to be representative of the Norwegian population with respect to age and gender, in addition to region. We excluded seven respondents who gave inconsistent answers (e.g., answering both “Never shopped online” and “Used online payment”), so the final sample consisted of 2202 adults.

### The Questionnaire

The questionnaire included 101 questions, the majority of which were designed by experts from the Consumer Finance Research Center (CFRC) in Rome, Italy, to be used for international comparisons of financial literacy and financial behaviour (Nicolini, 2019).^2^ The questionnaire was translated and adapted to the Norwegian market by a team of researchers and pretested by Kantar TNS to ensure that respondents understood the questions. The first part of the questionnaire asked about demographic information such as the respondents’ educational level, age, gender, income, and region. Part two included 50 financial literacy questions covering ten topics: interest rates, inflation, mortgages, investments, bonds, bank accounts, payments, savings and investment, loans and debts, and retirement and planning. There were five questions for each of the ten topics with increasing levels of difficulty. The third part included questions about various financial behaviours and preferences and the use of payment technologies. The fourth part asked about risk preferences and savings. All questions included the response options “do not know” and/or “prefer not to answer” and/or “not applicable” (NA).

## Measures

We analyze our data using binary logistics regressions. The variables used in the analysis are listed below.

### Dependent Variables

There are two sets of dependent variables: (1) use/nonuse of digital technological payment methods and attitudes toward using third parties (companies other than banks) for financial transactions and (2) indicators of financial vulnerability.

#### ***Use/Nonuse of Payment Technologies and Attitudes Toward Third Parties***

The respondents were asked if they use various types of payment technologies, asking them to check all the types of payment methods they are currently using. They were given examples for each payment method to clarify what was meant by each category. For the digital payment methods, these were online payment (e.g., PayPal or Klarna), mobile payment (e.g., Vipps, MobilePay, Mcash, Payr), and contactless payment (e.g., by holding the payment card over the terminal). For each of the three digital payment methods (mobile, online, and contactless payments), we constructed one dummy variable that equaled one if the respondent reported that he or she used the technology and zero if the respondent was a nonuser. Attitudes toward using third-party providers of financial services were measured by two questions: (1) “Are you willing to let your bank(s) share information about your account(s) with other companies if it would give you a better overview of your financials and make your financial services easier?” and (2) “If it were possible, would you be comfortable using a social media company (e.g., Facebook, Google or Twitter) to transfer money?”. The respondents could answer different versions of yes and no, and the answers were transformed into dummy variables where yes = 1 and no = 0.

#### Financial Vulnerability

The questionnaire included three questions that could tap into the degree of financial vulnerability of the respondents: (1) “How difficult do you find it to pay your bills in a usual month and to still have money left over for necessary expenditures?” (1 = “very difficult”, 2 = “difficult”, 3 = “a little bit difficult”, and 4 = “not at all difficult”). We coded the responses in two different ways: as an ordinal variable ranging from 1 to 4 (variable mean = 3.694, std. deviation = 0.644.) and as a dummy where all answers that conveyed some degree of difficulty in paying bills equaled 1 and “not at all difficult” equaled 0. (2) “In the last 5 years, did you ever use a credit line to buy some noninvestment goods, such as furniture, cars, TV screens, or cell phones, by using consumer credit?” (1 = Yes, 0 = No). The response categories and coding were the same as those for question 1. (3) “Do you have enough savings to cover 3 months of expenses in case you become ill or lose your job?” (1 = Yes, 0 = No). The response categories were yes/no, and a dummy variable where yes equals one was created.

### Independent Variables

#### Financial Literacy

The questionnaire contained 50 financial literacy questions that covered ten different financial topics. There were five questions per topic with various difficulties (see Nicolini, 2019). Five of these questions were equal to the so-called big five questions often used to measure financial literacy (Hastings et al., 2013). All 50 financial literacy items had two or three response options, in addition to “do not know” and “prefer not to say”.

Eight of the financial literacy questions were excluded from our analysis since they were either irrelevant for private investors in Norway or easily misunderstood by Norwegian consumers. These were four questions about bonds, one question about checks, and three questions where responses indicated that the questions or answers were misunderstood. An overall financial literacy score was constructed by summarizing the number of correct answers to the remaining 42 questions, giving the participants one point per correct answer. Only one answer was correct for each question. The score was used as a scale variable from 0 to 42 in most of the analysis, where a low score indicated a low level of financial literacy, and a high score indicated a high level of financial literacy. Variable mean = 24.459, std. deviation = 8.274. We also constructed an alternative ordinal variable as follows: low level of financial literacy (0–20 correct answers, 27.9% of N), medium level of financial literacy (21–30 correct answers, 45.9% of N) and high level of financial literacy (31–42 correct answers, 26.3% of N).

#### Generation

Age was measured in a scale variable (variable mean = 47.38, std. deviation = 17.04) and transformed into generation groups in line with Yakoboski et al. (2018) and Dimock (2019a, 2019b). The following definitions of generations were used: the Silent Generation (born 1928–1945), Baby Boomers (born 1946–1964), Generation X (born 1965–1980), Millennials (born 1981–1996), and Generation Z (born 1997–2012). Since, in our sample, only a small number of respondents belonged to Generation Z, this group was merged with the Millennials group.

#### Income

Household income was measured by the question “Approximately how large is the gross yearly income of your household (before taxes and deductions)?” with the following categories: “Below NOK 200,000” = 1, “NOK 200,000–399,999” = 2, “NOK 400,000–599,999” = 3, “NOK 600,000–799,999” = 4, “NOK 800,000–999,999” = 5, “NOK 1,000,000–1,199,999” = 6, “NOK 1,200,000–1,399,999” = 7, and “NOK 1,400,000 and above” = 8. Variable mean = 4.448, std. deviation = 1.881. NOK 1 was equal to USD 0.12 at the time the survey was conducted (DNB, 2019), thus NOK 200,000 = USD 24,000 for the lowest income category, and NOK 1.4 mill = USD 168,000 for the highest income category.

#### Education

The respondents were asked about their highest level of completed education. A dummy variable was created where those with some level of university or college education were defined as “highly educated” and coded = 1 and all other respondents were defined as “not highly educated” and coded = 0.

### Sample Characteristics

The sample characteristics are reported in Table 1. Men constitute 50.4% of the weighted sample. A majority of the sample is highly educated, and the ratio of highly educated respondents is almost the same for women and men. Approximately half of the sample reports having a medium income level. The respondents were asked to report their household income, and the average income reported by female respondents was lower than that reported by male respondents. Thus, when a woman is the respondent, more households are in the lowest income group than when the respondent is a man. This difference may be because the sample consists of many single-income households, and Norwegian women in general earn less than men. Another explanation may be that women who answer on behalf of a multiperson household are less aware of all of the income sources of their household and therefore underreport their household income. A third explanation could be that men and women tend to report income in line with the male breadwinner norm (Roth & Slotwinski, 2020).

**Table 1:** Sample characteristics (weighted)

		Gender		Generation group			
	Alle	Male	Female	Generation Z and Millennials (Born 1981–2012)	Generation X (Born 1965–1980)	Baby Boomers (Born 1946–1964)	Silent Generation (Born 1928–1945)
*N*	2202	1107	1095	793	541	701	167
**Use of digital payment methods**							
Online payment use	47.4%	45.8%	49.1%	60.7%	58.0%	32.8%	11.9%
Mobile payment use	67.9%	64.0%	71.9%	80.7%	76.3%	56.3%	29.6%
Contactless payment use	30.3%	32.1%	28.4%	40.5%	29.9%	22.8%	13.7%
**Financial vulnerability**							
Find it to some degree difficult to pay bills	21.6%	18.9%	24.3%	33.3%	24.5%	10.3%	4.7%
Have used CC or consumer debt to buy consumable goods	21.5%	23.0%	19.9%	23.7%	27.6%	16.5%	11.8%
Have saved up for 3 months of exp	67.5%	71.0%	64.0%	54.9%	63.4%	80.1%	86.4%
**Attitudes to third party providers**							
Willing to let bank share customer information	28.9%	32.9%	24.7%	37.2%	28.3%	21.1%	25.0%
Unwilling to let bank share customer information	55.9%	56.4%	55.4%	46.1%	55.2%	65.5%	62.2%
Willing to use social media for money transfer	14.8%	19.1%	10.6%	22.9%	13.5%	9.0%	5.4%
Unwilling to use social media for money transfer	74.5%	71.3%	77.8%	65.7%	72.3%	83.0%	86.9%
**Level of financial literacy**							
Low (0–20 correct answers)	27.9%	15.6%	40.3%	32.2%	28.8%	22.5%	26.8%
Medium (21–30 correct answers)	45.9%	44.9%	46.8%	44.8%	41.4%	48.9%	52.4%
High (31–42 correct answers)	26.3%	39.5%	12.9%	22.9%	29.8%	28.5%	20.8%
**Gross household income**							
NOK < 400 K	13.6%	10.3%	16.9%	24.9%	9.4%	6.3%	10.6%
NOK 400 K – 999.999	52.6%	55.5%	49.8%	50.2%	43.6%	57.3%	73.1%
NOK > 1 M	24.3%	27.2%	21.5%	24.9%	39.1%	26.0%	7.8%
**Education level**							
Higher education	60.3%	59.1%	61.5%	62.3%	62.5	56.7%	59.4%

Table 1 also shows that the same pattern of financial literacy levels found in other countries is also found in Norway, e.g., women have significantly lower financial literacy scores than men (Bucher-Koenen et al., 2017; Lusardi & Mitchell, 2011a, 2011b, Mahdavi & Horton, 2014; Scheresberg et al., 2020; Stolper & Walter, 2017). A total of 40.3% of women were classified as having low financial literacy, while this was only the case for 15.6% of men. On the other hand, 39.5% of men are classified as having high financial knowledge, compared to only 12.9% of women. A further inspection of the data shows that the tendency to answer “I don’t know” is considerably higher among women than men. The table also shows that among the generation groups, Millennials (born 1981–1996) have the highest ratio of respondents with low financial literacy scores, while they, together with the Silent Generation (born 1928–1945), have the lowest ratio of respondents with high literacy scores. Hence, in Norway, we find that women and young people are the least financially literate, which may make them more financially vulnerable. The highest level of financial literacy is found among Baby Boomers (born 1946–1964).

Regarding financial vulnerability, Table 1 reveals that approximately the same percentage of participants finds it to some degree difficult to pay the bills (21.6%) and have used consumer credit to buy consumable goods (21.5%). However, this is distributed somewhat differently between different groups, where more women (24.3%) than men (18.9%) find it difficult to pay the bills, but more men (23.0%) than women (19.9%) have used consumer credit. We also see that the younger generations have a higher percentage of these vulnerability variables than the older generations. For the third vulnerability variable, having saved up for 3 months of expenses, 67.5% of the participants reported that they had done so. This was more common for men (71.0%) than women (64.0%), and the older generations were more likely to have saved than the younger generations.

In regard to payment technologies, Table 1 reveals that the use of mobile payments is more frequent among women (71.9%) than men (64.0%), which is the opposite of findings from the US population (Lusardi et al., 2017; Meyll & Walter, 2019; Scheresberg et al., 2020; Yakoboski et al., 2018). A similar result is found for online payments, where 49.1% of women and 45.8% of men report usage. On the other hand, men are found to be more frequent users of contactless payments, which are used by 32.1% of men compared to 28.4% of women (however, a chi-square test on gender and contactless payments finds that this difference is not significant). As expected, younger generations are more frequent users of all three digital payment methods than older generations. Among Millennials (born 1981–1996), 80.7% use mobile payments, 40.5% use contactless payments, and 60.7% use online payments; this user pattern is in stark contrast to that of the Silent Generation (born 1928–1945), for which the corresponding numbers are 29.6%, 13.7%, and 11.9%, respectively.

## Results

### Use/Nonuse of Payment Technologies and Attitudes Towards Third Parties

Table 2 reports the results of binary logistic regression analyses where the use of payment technologies and attitudes toward third parties were dependent variables. Participants choosing the response options “prefer not to answer” and/or “NA” on any of the questions were excluded from the analyses. The impact of the probability of using the different technologies was tested on all control variables (financial literacy, gender, generation group, household income, and educational level) to determine who the probable users of the various payment technologies were. The linearity of the continuous variables “financial literacy score” and “gross household income” with respect to the logit of the dependent variable was assessed via the Box and Tidwell (1962) procedure. All continuous independent variables were found to be linearly related to the logit of the dependent variables.

**Table 2:** Results of binary logistic regression analyses of use of three digital payment methods and attitudes towards using third parties for financial transactions

	Online payment	Mobile payment	Contactless payment	Willing to let bank share customer information	Willing to use social media for money transfer
Financial literacy score	1.052***	1.094***	1.073***	1.004	1.031*
	**(6.375)**	**(10.000)**	**(7.889)**	(0.400)	**(2.583)**
Female	1.426***	2.572***	1.258	0.821	0.598***
	**(3.114)**	**(7.159)**	(1.901)	(− 1.576)	**(**− **3.179)**
Generation X (Born 1965–1980)	0.880	0.567***	0.493***	0.548***	0.478***
	(0.914)	**(**− **3.203)**	**(**− **4.738)**	**(**− **3.909)**	**(**− **3.931)**
Baby Boomers (Born 1946–1964)	0.306***	0.189***	0.373***	0.363***	0.308***
	**(**− **8.828)**	**(**− **10.354)**	**(**− **6.895)**	**(**− **6.812)**	**(**− **6.266)**
Silent Generation (Born 1928–1945)	0.081***	0.075***	0.236***	0.388***	0.138***
	**(**− **8.440)**	**(**− **10.996)**	**(**− **5.381)**	**(**− **3.983)**	**(**− **4.605)**
Gross income for the household	1.021	1.206***	1.095**	1.060*	1.045
	(0.677)	**(5.054)**	**(2.758)**	**(1.735)**	(1.073)
Highly educated	1.270**	1.145	1.195	1.192	1.281
	**(2.096)**	(1.079)	(1.435)	(1.386)	(1.521)
Constant	0.287***	0.174***	0.067***	0.629	0.145***
	**(**− **5.136)**	**(**− **6.353)**	**(**− **9.525)**	(− 1.611)	**(**− **5.270)**
Observations	1673	1673	1673	1420	1492
R-squared (Nagelkerke)	0.175	0.278	0.123	0.060	0.101

Note: The first three dependent variables are online payment usage, mobile payment usage, and contactless payment usage based on the question “Which of the following payment solutions do you use?” and the following answers: “online payments”, “mobile payments,” and “contactless payments”. Responses were coded 1 if the respondents answered that they use the payment method and zero if they indicated they do not. The two last dependent variables are based on the questions “Are you willing to let your bank(s) share information about your accounts with other companies if it would give you a better overview of your financials and make your financial services easier?” and “If it were possible, would you be comfortable using a social media company (e.g., Facebook, Google or Twitter) to transfer money?”. The respondents could answer yes/no, and the answers were transformed into a dummy variable where yes = 1 and no = 0. Respondents who indicated that they did not know or preferred not to say were excluded. Baseline categories: male, Generation Z + Millennials (born 1981–2012), and not highly educated. The table reports odds ratios. T values are in parentheses. *** *p* < 0.001, ** *p* < 0.01, * *p* < 0.05

For online payments, the logistic regression model was statistically significant, *χ*^2^(7) = 233.104, *p* < 0.001. The model explained 17.5% (Nagelkerke *R*^2^) of the variance in participants’ use of online payment methods and correctly classified 65.8% of cases. The probability of someone using online payments increased with higher financial literacy scores, with an odds ratio of 1.052. Women had 1.426 times higher odds of using online payments than men. Additionally, compared to Millennials/Generation Z (born 1981–2012), both Baby Boomers (born 1946–1964) and those in the Silent Generation (born 1928–1945) were less likely to use online payment, with odds ratios of 0.306 and 0.081, respectively. Regarding education, there was a higher probability of use among those with a higher educational level, with an odds ratio of 1.270.

Similar results were found for both mobile payments and contactless payments. The logistic regression model for mobile payment was statistically significant, *χ*^2^(7) = 364.536, *p* < 0.001, explained 27.8% (Nagelkerke *R*^2^) of the variance in participants’ use of mobile payment methods and correctly classified 75.3% of cases. For contactless payments the model was also statistically significant, *χ*^2^(7) = 150.628, *p* < 0.001, explaining 12.3% (Nagelkerke *R*^2^) and correctly classifying 69.7% of cases. The probability of using mobile payments increased with higher financial literacy scores, with an odds ratio of 1.094. Women had 2.573 times higher odds of using mobile payments than men. Generation X (born 1965–1980), Baby Boomers (born 1946–1964), and those in the Silent Generation (born 1928–1945) were less likely to use mobile payment than the Millennials/Generation Z (born 1981–2012), with odds ratios of 0.567, 0.189, and 0.075, respectively. The probability of using contactless payments also increased with higher financial literacy scores (odds ratio of 1.073). Those belonging to Generation X (born 1965–1980), Baby Boomers (born 1946–1964), and those in the Silent Generation (born 1928–1945) were less likely to use contactless payment than Millenials/Generation Z (born 1981–2012), with odds ratios of 0.494, 0.373, and 0.236, respectively.^3^

### Allowing Payment Providers Access to Account Data

Table 2 also reports the results of a logistic regression analyses of the variables related to letting banks share information with a third party. The logistic regression model was statistically significant, *χ*^2^(7) = 61.919, *p* < 0.001, explained 6.6% (Nagelkerke *R*^2^) of the variance, and correctly classified 65.3% of cases. The probability of being willing to let their bank share information with third-party companies was higher among younger generations than among older generations, with odds ratios of 0.548 for those belonging to Generation X (born 1965–1980), 0.363 for Baby Boomers (born 1946–1964), and 0.388 for those in the Silent Generation (born 1928–1945) compared to Millennials/Generation Z respondents (born 1981–2012). Level of financial literacy, education, and gender were not significant predictors of willingness to share account data.

### Willingness to Use a Social Media Company for Money Transfers

The logistic regression model for willingness to use a social media company for money transfers was statistically significant, *χ*^2^(7) = 90.287, *p* < 0.001, explained 10.1% (Nagelkerke *R*^2^) of the variance, and correctly classified 83.7% of cases. The probability of being willing to use a social media company for money transfers increased with higher financial literacy scores, with an odds ratio of 1.031. Compared to men, women were less willing to engage in this behaviour, with an odds ratio of 0.598. Once again we find the younger generation to be more positive to digital innovations, and the probability of being willing to use social media for money transfers was higher among younger generations than among older generations (odds ratios of 0.478 for those belonging to Generation X (born 1965–1980), 0.308 for Baby Boomers (born 1946–1964), and 0.138 for those in the Silent Generation (born 1928–1945) compared to Millennials/Generation Z respondents (born 1981–2012)).

## Financial Vulnerability

Table 3 reports the results from binary logistic regression analyses using the three financial vulnerability variables as dependent variables and digital payment methods as predictor variables. Participants who answered the three vulnerability questions by choosing the response options “prefer not to answer” and/or “NA” were excluded from the analyses. We controlled for financial literacy, gender, generation group, household income, and educational level and tested whether there were any interaction effects between generation groups and the use of digital payment methods. The linearity of the continuous variables with respect to the logit of the dependent variable was assessed via the Box and Tidwell (1962) procedure, and all continuous independent variables were found to be linearly related to the logit of the dependent variables.

**Table 3:** Results of binary logistic regression analyses of financial vulnerability

	Find it difficult to pay bills	Have used CC or consumer debt to buy consumable goods	Have saved for 3 months of exp
Financial literacy score	0.943***	0.989	1.049***
	**(**− **5.364)**	(− 0.110)	**(4.800)**
Female	0.955	0.724*	1.025
	(− 0.293)	**(2.307)**	(0.179)
Generation X (Born 1965–1980)	0.429*	0.837	0.876
	**(**− **2.161)**	(− 0.471)	(− 0.338)
Baby Boomers (Born 1946–1964)	0.115***	0.375**	2.186*
	**(**− **6.099)**	**(**− **2.952)**	**(2.280)**
Silent Generation (Born 1928–1945)	0.052***	0.322**	12.516***
	**(5.694)**	**(**− **2.842)**	**(4.163)**
Gross household income	0.765***	1.078*	1.146***
	**(**− **6.091)**	**(1.974)**	**(3.579)**
Highly educated	1.033	0.665**	0.937
	(0.209)	**(**− **3.022)**	(− 0.471)
Users of online payments	0.866	1.314	0.819
	(− 0.548)	(1.019)	(− 0.823)
Users of mobile payments	0.395**	0.591	0.731
	**(**− **2.870)**	(− 1.594)	(− 0.949)
Users of contactless payments	1.208	1.059	0.771
	(0.724)	(0.222)	(− 1.102)
Users of online payments X Generation X	1.095	1.237	1.019
	(0.247)	(0.591)	(− 0.056)
Users of online payments X Baby Boomers	2.109	1.634	0.763
	(1.824)	(1.387)	(− 0.772)
Users of online payments X Silent Generation	0.000	0.650	0.386
	(− 0.002)	(− 0.509)	(− 1.018)
Users of mobile payments X Generation X	3.602**	1.119	1.025
	**(2.862)**	(0.259)	(0.057)
Users of mobile payments X Baby Boomers	2.622*	1.486	1.445
	**(2.176)**	(0.983)	(0.889)
Users of mobile payments X Silent Generation	3.967	1.527	0.540
	(1.687)	(0.672)	(− 0.744)
Users of contactless payments X Generation X	0.478	1.064	1.553
	(− 1.859)	(0.173)	(1.275)
Users of contactless payments X Baby Boomers	0.428	0.614	1.361
	(− 1.715)	(− 1.281)	(0.813)
Users of contactless payments X Silent Generation	0.716	0.953	2.342
	(− 0.292)	(− 0.064)	(0.722)
Constant	10.957***	0.637	0.505
	**(5.940)**	(− 1.232)	(− 1.819)
Observations	1624	1626	1593
R-squared (Nagelkerke)	0.235	0.068	0.177

Note: The first dependent variable is based on the question “How difficult do you find it to pay your bills in a usual month and to still have money left over for necessary expenditures?”. The response options were “very difficult” = 1, “difficult” = 2, “a little bit difficult” = 3, and “not at all difficult” = 4. We coded the responses as a dummy where all answers that conveyed some degree of difficulty paying bills equaled one and “not at all difficult” equaled zero. Respondents who indicated that they did not know or preferred not to say were excluded. The second and third dependent variables are based on the following questions: “In the last 5 years, did you ever use a credit line to buy some noninvestment goods, such as furniture, cars, TV screens, or cell phones, by using consumer credit?” and “Do you have enough savings to cover 3 months of expenses in case you become ill or lose your job?”. The response categories were yes/no. A dummy variable where yes = 1 and *n* = 0 was created. Respondents who indicated that they did not know or preferred not to say were excluded. Baseline categories: Male, Generation Z + Millennials (born 1981–2012), not highly educated, nonuser of online payments, nonuser of mobile payments, and nonuser of contactless payments. The table reports odds ratios. T values are in parentheses. *** *p* < 0.001, ** *p* < 0.01, * *p* < 0.05

### Difficulty with Paying Bills

The logistic regression model for having difficulty paying bills was statistically significant, *χ*^2^(19) = 249.574, *p* < 0.001, explained 23.5% (Nagelkerke *R*^2^) of the variance, and correctly classified 82.9% of cases. The probability of someone finding it difficult to pay their bills decreased as the financial literacy score increased, with an odds ratio of 0.943. Compared to Millennials/Generation Z (born 1981–2012), the odds ratio of having difficulty paying bills was 0.429 for those belonging to Generation X (born 1965–1980), 0.115 for Baby Boomers (born 1946–1964) and 0.052 for those in the Silent Generation (born 1928–1945). As expected, a higher income is associated with a lower probability of payment problems (odds ratio = 0.765). In contrast to the findings from the USA, we found that mobile payment users are significantly less likely to find it difficult to pay their bills, with an odds ratio of 0.395 compared to nonusers. We did not find use of online or contactless payment methods to predict payment problems.

Regarding interaction effects overall, there were statistically significant relationships between generation groups and the use of mobile payments. Adding the interaction effects in block 2 of the analysis improved the explanatory power of the model from a Nagelkerke *R* square value of 0.219 to 0.235. A positive interaction effect was found between mobile payment users and those belonging to Generation X (born 1965–1980) (odds ratio = 3.602) and Baby Boomers (born 1946–1964) (odds ratio = 2.622). The rest of the interaction effects were not significant. These results show that relationships between payment method and financial vulnerability differ somewhat between generation groups.

### Use of Consumer Credit to Buy Consumable Goods

As for the use of consumer credit to buy consumable goods, the logistic regression model was statistically significant, *χ*^2^(19) = 73.248, *p* < 0.001, explained 6.8% (Nagelkerke *R*^2^) of the variance, and correctly classified 78.7% of cases. The results showed that men are more likely than women to have used consumer credit to buy consumable goods (odds ratio of 0.724). The probability of using credit for consumable goods was lower for Baby Boomers (born 1946–1964) (odds ratio = 0.375) and respondents belonging to the Silent Generation (born 1928–1945) (odds ratio = 0.322) compared to Millennials/Generation Z respondents (born 1981–2012). We found that the probability of using consumer credit increased with gross household income (odds ratio = 1.078) and decreased with higher education level (odds ratio = 0.665). None of the interaction terms were found significant in this analysis although the inclusion of the interaction terms improved the explanatory power of the model, with the Nagelkerke *R* square value changing from 0.063 in block 1 to 0.068 in block 2. In this extended model, the payment methods were no longer significant.

Looking at the different generations separately, we see that only the Silent Generation (born 1928–1945) showed a significant result regarding financial literacy. In this group, the probability of using consumer credit to buy consumable goods decreased with increasing financial literacy scores (odds ratio = 0.896). Gender showed a significant effect only among Baby Boomers (born 1946–1964), where women were less likely than men to have used consumer debt, with an odds ratio of 0.443. Household income was an important predictor for use of consumer credit among Millennials/Generation Z respondents (born 1981–2012), where an increase in income increased the likelihood of using consumer debt (odds ratio = 1.211). Among online payment users, both Generation X respondents (born 1965–1980) and Baby Boomers (born 1946–1964) were found to be more likely to have used consumer loans than nonusers, with odds ratios of 1.689 and 2.112, respectively. However, looking at mobile payment users, we found that Millennials/Generation Z respondents (born 1981–2012) were less likely to have used consumer loans (odds ratio = 0.500).

### Saved Enough for at Least Three Months of Expenses

A logistic regression was performed to ascertain the effects of digital payment methods on the likelihood that someone had saved up enough money to cover at least three months of expenses. The model was statistically significant, *χ*^2^(19) = 199.206, *p* < 0.001, explained 17.7% (Nagelkerke *R*^2^) of the variance, and correctly classified 77.1% of cases. The probability of having saved up three months of expenses increased with higher financial literacy scores (odds ratio = 1.049). The two oldest generations were more likely than the Millennials/Generation Z respondents (born 1981–2012) to have saved at least three months of expenses, with odds ratios of 2.186 for Baby Boomers (born 1946–1964) and 12.516 for the Silent Generation (born 1928–1945). As expected, the probability of having saved at least three months of expenses increased with income (odds ratio = 1.146). The use of digital payment technologies did not predict having this saving buffer. The interaction terms were not found to be significant predictors of having saved three months of expenses; however, the inclusion of the interaction terms in the model significantly improved its explanatory power, with the Nagelkerke *R* square value changing from 0.173 in block 1 to 0.177 in block 2.

Looking at the four generations separately, we found a positive relationship between financial literacy and having saved up enough money to cover at least three months of expenses among those belonging to Generation X (born 1965–1980), Baby Boomers (born 1946–1964), and those in the Silent Generation (born 1928–1945) (odds ratios 1.057, 1.063, and 1.135, respectively). We also found significant gender differences. Compared to men in the same generation, Millennial women were less likely to have saved, whereas female Baby Boomers (born 1946–1964) were more likely, with odds ratios of 0.444 and 1.924, respectively. Among Millennials/Generation Z respondents (born 1981–2012) (odds ratio = 1.146) and those belonging to Generation X (born 1965–1980) (odds ratio = 1.213), an increase in household income increased the probability of having saved up at least three months of expenses. There were no significant relationships between payment methods and saving.

### Difficulty with Paying Bills—by Generation Group

To further study the differences between the generation groups, binary logistic regressions were run separately for each group (Table 4). Except for the Silent Generation (born 1928–1945), which had no significant results, we found a negative relationship between financial literacy and having difficulty paying bills in all generation groups, with odds ratios of 0.995 for Millennials/Generation Z (born 1981–2012), 0.894 for Generation X (born 1965–1980), and 0.950 for Baby Boomers (born 1946–1964). Considering digital payment methods, mobile payment users among Millennials/Generation Z respondents (born 1981–2012) were less likely to have difficulty paying their bills (odds ratio = 0.345) compared to the rest of the participants.

**Table 4:** Binary regression analyses of financial vulnerability across generation groups

	Find it difficult to pay bills	Have used CC or consumer debt to buy consumer goods	Have saved for 3 months of exp	Find it difficult to pay bills	Have used CC or consumer debt to buy consumer goods	Have saved for 3 months of exp	Find it difficult to pay bills	Have used CC or consumer debt to buy consumer goods	Have saved for 3 months of exp	Find it difficult to pay bills	Have used CC or consumer debt to buy consumer goods	Have saved for 3 months of exp
	Generation Z and Millennials (Born 1981–2012)			Generation X (Born 1965–1980)			Baby Boomers (Born 1946–1964)			Silent Generation (Born 1928–1945)		
Financial literacy score	0.955**	1.003	1.025	0.894***	0.989	1.057**	0.950*	0.997	1.063***	1.000	0.896**	1.135*
	**(**− **2.556)**	(0.167)	(1.471)	**(**− **5.091)**	(− 0.647)	**(3.056)**	**(**− **2.429)**	(0.167)	**(3.588)**	(0.007)	**(**− **2.659)**	**(2.190)**
Female	1.313	1.244	0.444***	0.795	0.746	1.331	0.670	0.443***	1.924**	1.483	0.760	0.921
	(1.023)	(0.787)	**(**− **3.180)**	(− 0.734)	(− 1.149)	(1.113)	(− 1.302)	**(**− **3.273)**	**(2.569)**	(0.493)	(− 0.503)	(− 0.104)
Gross household income	0.909	1.211**	1.146*	0.587***	0.940	1.213**	0.707***	1.096	1.073	0.579	1.152	1.286
	(− 1.439)	**(2.851)**	**(2.141)**	**(**− **6.213)**	(− 0.939)	**(2.881)**	**(**− **3.541)**	(1.314)	(0.946)	(− 1.599)	(− 0.667)	(0.696)
Highly educated	1.142	0.711	0.960	0.970	0.670	0.954	1.022	0.505**	0.967	2.142	2.547	0.277
	(0.524)	(− 1.302)	(− 0.168)	(− 0.106)	(− 1.610)	(− 0.187)	(0.070)	**(**− **2.931)**	(− 0.137)	(0.990)	(1.664)	(− 1.361)
Users of online payments	0.857	1.265	0.892	1.051	1.689*	0.829	1.759	2.112**	0.651	0.000	0.894	0.239
	(− 0.603)	(0.867)	(− 0.458)	(0.167)	**(2.113)**	(− 0.764)	(1.766)	**(3.169)**	(− 1.686)	(− 0.002)	(− 0.133)	(− 1.439)
Users of mobile payments	0.345***	0.500*	0.759	1.923	0.716	0.691	1.102	0.929	0.974	1.293	0.871	0.380
	**(**− **3.284)**	**(**− **2.029)**	(− 0.804)	(1.772)	(− 1.113)	(− 1.190)	(0.311)	(− 0.302)	(− 0.101)	(0.334)	(− 0.254)	(− 1.168)
Users of contactless payments	1.133	1.017	0.792	0.516	1.176	1.376	0.521	0.633	1.057	0.660	1.251	1.422
	(0.486)	(0.064)	(− 0.959)	(− 1.870)	(0.616)	(1.190)	(− 1.538)	(− 1.598)	(0.187)	(− 0.366)	(0.310)	(0.293)
Constant	3.927*	0.251*	1.219	40.159***	0.909	0.254**	1.670	0.240**	0.814	0.218	0.739	1.866
	**(2.447)**	**(**− **2.474)**	(0.356)	**(5.344)**	(− 0.187)	**(**− **2.600)**	(0.829)	**(**− **2.750)**	(− 0.405)	(− 0.905)	(− 0.279)	(0.431)
Observations	372	373	368	395	399	387	670	668	656	177	176	172
R-squared (Nagelkerke)	0.126	0.048	0.112	0.354	0.043	0.102	0.101	0.091	0.055	0.105	0.101	0.194

Note: This table lists the results of binary regressions run separately for each generation group. The first dependent variable is based on the question “How difficult do you find it to pay your bills in a usual month and to still have money left over for necessary expenditures?”. The response options were “very difficult” = 1, “difficult” = 2, “a little bit difficult” = 3, and “not at all difficult” = 4. We coded the responses as a dummy where all answers that conveyed some degree of difficulty paying bills equaled one and “not at all difficult” equaled zero. Respondents who indicated that they did not know or preferred not to say were excluded. The second and third dependent variables are based on the questions “In the last 5 years, did you ever use a credit line to buy some noninvestment goods, such as furniture, cars, TV screens, or cell phones, by using consumer credit?” and “Do you have enough savings to cover 3 months of expenses in case you become ill or lose your job?”. The response categories were yes/no. A dummy variable where yes = 1 and *n* = 0 was created. Respondents who indicated that they did not know or preferred not to say were excluded. Baseline categories: Male, Generation Z + Millennials (born 1981–2012), not highly educated, nonuser of online payments, nonuser of mobile payments, and nonuser of contactless payments. The table reports odds ratios. T values are in parentheses. *** *p* < 0.001, ** *p* < 0.01, * *p* < 0.05

In summary, Generation X (born 1965–1980) participants using mobile payments were more likely to find it difficult to pay their bills, contactless payment users were less likely to find it difficult, and online payment users were more likely to have used consumer debt to buy consumable goods. Our hypothesis that the users of existing digital payment technologies would most likely be men was contrary to what we found. Additionally, in contrast to our hypothesis, adopters and users of digital payment technologies were found to have higher financial literacy than nonusers. Mobile payment users were also less likely to find it difficult to pay their bills. Breaking down these results by generation, we found that there was a difference between the groups. Millennial mobile payment users were found to be less financially vulnerable than nonusers. However, among Baby Boomers (born 1946–1964), online payment users were more financially vulnerable than nonusers. Regarding the use of social media companies for money transfers, men were more likely to be adopters. In line with our hypothesis, the users/adopters of digital payment methods were more likely to be highly educated, to be high earners, and to belong to younger generations.

## Discussion

### Use/Nonuse of Payment Technologies and Attitudes Toward Using Third Parties

With respect to the effects of financial literacy, we found a positive relationship between the use of any of the three payment methods and a higher financial literacy score. This relationship was significant both when using the score on the 42 financial knowledge questions and when using the Big 3 and Big 5 questions often employed to measure financial literacy (Hastings et al., 2013; Huston, 2010). Hence, in this study, we found that users of the new digital financial payment methods have higher financial literacy than nonusers, which is in contrast to the results of US studies (Liao & Chen, 2020; Lusardi et al., 2017; Scheresberg et al., 2020; Yakoboski et al., 2018). Similar relationships were found regarding the respondents’ willingness to use social media companies for payment services. The reasons for the differences in results between countries may be the generally higher level of financial literacy in Norway, where 71% of adults were found to be financially literate compared to 57% of Americans according to a financial literacy survey conducted by Klapper and Lusardi (2020). The high level of digitalization could also play a role.

Compared to men, women were found to be more likely users of both online and mobile payment methods. This finding is surprising considering that several previous studies have found the opposite to be true with respect to mobile payments (Gerpott & Meinert, 2017; Lusardi et al., 2017; Meyll & Walter, 2019; Scheresberg et al., 2020; Yakoboski et al., 2018). These differences in results may be because the survey was conducted in one of the most digitally advanced and most gender-equal countries worldwide (WEF, 2016, 2020), making it likely that Norwegian women are more digitally advanced than women in countries such as the USA.

Something that can also be seen as surprising was that men were more likely to be willing to use social media companies for money transfers than women. Considering that women are more active on social media than men (Greenwood et al., 2016; Khoros, 2021), one might expect them to also be more open to using social media for payments. However, the fact that we see the opposite here could be an indication that men are more likely first adopters of this type of technology. On the other hand, we do see indications that women are more likely to use payment technologies when the initial first adoption face has passed.

Not surprisingly, younger generations were found to be more likely users of digital payment methods. This result is as expected considering that younger generations are more used to digital tools than older generations. However, we should also consider the possibility that openness to using social media companies for money transfers could be related to the younger generation’s lower financial literacy and lack of knowledge about security issues related to various payment technologies.

### Financial Vulnerability

In line with the results of previously mentioned studies, participants with higher financial literacy scores were less likely to find it difficult to pay their bills and were more likely to have saved up enough money to cover 3 months of expenses. This relationship is an indication that having a basic understanding of financial literacy is helpful with regard to keeping track of personal finances. In contrast to the findings of the studies by Yakoboski et al. (2018), Meyll and Walter (2019), and Scheresberg et al. (2020), we found that the use of mobile payments was unrelated to problematic financial behaviour. In fact, we found that users of mobile payments as a whole were less likely to have difficulty paying their bills. This is a promising result, as an increasing number of countries are moving away from cash and toward digital payment. The results could indicate that digital payment methods may not have as much of a worrying effect as was found in US studies, at least not in all countries.

In our study, the use of digital payment methods was linked to more behaviour that reduces vulnerability than what was found in previous studies (Meyll & Walter, 2019; Scheresberg et al., 2020; Yakoboski et al., 2018). A possible explanation for this could be that the consumers in our study have had more time to adapt to these kinds of payment methods and are therefore more used to them. Adaptation time could perhaps be an important variable in future studies. In any case, it emphasizes that the results from one country regarding the effects of digital payment methods cannot be generalized to other countries.

We observed that the youngest generations (Gen Z. and Mill) were more likely to have difficulty paying their bills and to have used consumer debt to buy consumable goods, along with being less likely to have saved up for a rainy day compared to the other generations. This could be construed as worrying. It is likely that this is related to the fact that income levels increase with age and that younger generations typically have higher long-term debt (e.g., mortgages and student loans) than older generations. That being said, they may also be linked to younger generations having lower levels of financial literacy. The younger generations also had more debt, which is not surprising. However, it shows that the lower income levels among younger generations make them more vulnerable to economic shocks, and for this reason, it is important that young people have the necessary skills to deal with unexpected financial events.

## Conclusion

This study examined the characteristics and financial behaviour of users of various digital payment methods and whether the adoption of these methods can be linked to different types of financial vulnerability. We also investigated whether any group is less financially knowledgeable than others and, if so, how lack of knowledge is reflected in financial behaviour and attitudes toward using new providers of payment services. Although more research on this topic has been in demand for the last few years, it has rapidly become more important due to the growing shift to online spending that came with COVID-19.

Our primary expectations were that users of new payment technologies would be more financially vulnerable and have lower financial literacy than nonusers and that they were likely to be men, highly educated, and high earners and to belong to younger generations. Some of these expectations were supported. However, with regard to financial vulnerability, gender, and financial literacy, our findings deviated from our hypotheses. The use of mobile payments was found not to be related to problematic financial behaviour, and users were in fact less likely to have difficulty paying their bills than nonusers. We need to acknowledge that more comprehensive measures of the vulnerability variables would have improved the study, for example, by distinguishing between short-term and long-term financial problems. It is more worrying if a consumer has chronic financial difficulties than if he or she has problems paying a bill once or twice a year. Future studies can preferably add more vulnerability variables.

In contrast to the findings of previous scholars, (Lusardi et al., 2017; Meyll & Walter, 2019; Scheresberg et al., 2020), we found that women were more likely than men to be users of digital payment technologies. In addition, the probability of using these technologies actually increased with the increase in the financial literacy score. A weakness of this study is that it is cross-sectional and does not allow causal interpretations. Therefore, we do not know whether, for example, high financial literacy causes the increased use of digital payment methods or whether the use of different payment methods enhances financial literacy. However, these results show that previous results from the USA cannot be generalized to Northern European countries, which underlines the necessity of more studies from outside the USA. We do, however, find it reassuring that others have found higher financial literacy to reduce the negative effects of using mobile payments (Lusardi et al., 2017; Yakoboski et al., 2018), as our findings show that the use of digital payments becomes more commonplace as financial literacy increases. Consumers who understand finance will be less likely to let new payment technologies negatively affect their personal finances.

Finally, we found that first adopters of new financial payment technologies likely have higher financial literacy scores and belong to younger generations. Different from the use of digital payment methods, men were found to be more willing to use social media companies for money transfers, indicating that men are more often first adopters, whereas women are more likely to be users once the use of payment technologies has become more commonplace.

From the results above, we do not find a reason to be worried regarding the changes introduced relating to the new EU payment directive (PSD2). Nonetheless, now that the directive has come into effect in Europe and many changes to financial systems have occurred, more research should be conducted to establish whether people truly are as skeptical about these changes as they indicated to us before anything came into effect.

## Implication for Policy

As we have argued previously, the world is moving quickly toward forsaking cash for digital financial tools, and the ongoing COVID-19 pandemic has been an accelerator of this change. With this study, we have sought to gain more knowledge about the use of digital financial tools and financial vulnerability in the hope that this could be of help to stifle financial mismanagement.

The diverging results of the US studies and our Northern European study are interesting. Although similar investigations should be conducted in additional countries, the available studies may offer lessons to learn for policy makers and regulators. The results indicate that digital payment methods may lead to financial difficulties when users do not possess the necessary digital and financial competencies to use them in a rational manner. This lack of competencies may be one possible explanation for why the use of mobile payments is associated with financial problems in the USA since the general level of financial literacy and digitalization is lower there than in Northern Europe. It may also partly explain why we find use of certain digital payment methods to be associated with behaviour that could increase financial vulnerability among those belonging to Generation X (born 1965–1980) and Baby Boomers (born 1946–1964) but not among Millennials/Generation Z (born 1981–2012). These younger generations have more experience with technology than older generations and may therefore be better at using digital tools to control their spending.

There needs to be a consideration that the younger generations’ openness to the use of social media companies for money transfers could be related to their lack of knowledge about security issues related to some of these payment technologies. Social media companies are important market communication channels, and giving providers of sales promotions access to members’ financial data may enable better targeted and more persuasive market offers. Combined with lower financial literacy and a less transparent payment method, this factor may increase the difficulty that people have in keeping track of and controlling their expenses. This again might be part of the explanation as to why Millennials/Generation Z (born 1981–2012) were found to have more worrying financial management than all the other generations.

We see from our findings that consumers who understand finance seemed less likely to let new payment technologies negatively affect their personal finances. A natural progression to this finding is to ask whether the level of financial literacy could be increased through financial education or some form of “just-in-time” education tied specifically to the use of fintech-like digital payment methods (Fernandes et al., 2014). Either way, higher levels of financial literacy are found to correlate with lower financial vulnerability, so focusing on educating the population on relevant financial issues could be seen as a good investment for the future. Considering the increased speed of change we see in the world of financial technology today; this should probably be done sooner rather than later.

## Data Availability

The data that support the findings of this study are available from the corresponding author upon reasonable request. After publication of the study, the data will be submitted to NSD archives of research data (https://www.nsd.no/en/archiving-research-data).

## Footnotes

1 In 2016, Norway was ranked 4th (the USA was ranked 5th) in the World Economic Forum’s Network Readiness Index, which measured how well the economy uses technologies to boost competitiveness and well-being WEF (2016). Networked Readiness Index. Networked Readiness Index. World Economic Forum http://reports.weforum.org. In 2019, 98% of Norwegian households had internet access, and 95% of the population had access to a smartphone SSB, S. N. (2020). Fakta om Internett og mobil. https://www.ssb.no/teknologi-og-innovasjon/faktaside. Accessed 12 Oct 2020.

2 The questionnaire was developed by experts from the Consumer Finance Research Center (CFRC) in Rome, Italy, to analyze the financial literacy and financial behaviour of European financial consumers. The questionnaire was built on previous studies, and the questions were customized to fit each participating country. The project is described in Nicolini (2019) and at http://www.consumer-finance.org/CFRC/Research.htm

3 The analyses were rerun using alternative measures of financial literacy. When using the so-called Big 3 and Big 5 as measures of financial literacy, we found the same results as when using the total score: Use of digital technologies increases with higher financial literacy scores. When using 10 different scores, one for each of the financial topics (accounts, savings and bonds, interest, diversification, effective interest, inflation, mortgage, and retirement), we found few significant relationships between financial literacy and use of technology. However, the probability of use of online payments and contactless payments increased significantly with an increase in correct answers about accounts (odds ratio = 1.477 for online payments and = 1.530 for mobile payments). The same results were found with the number of correct answers about payments; higher knowledge about payments significantly increased the probability of use of online and contactless payment methods (odds ratios of 1.234 (online payments) and 1.411 (mobile payments)). In addition, there was a higher probability of use of online payments with the increase in correct answers about APR, with an odds ratio of 1.186.
